# Conservation laws by virtue of scale symmetries in neural systems

**DOI:** 10.1371/journal.pcbi.1007865

**Published:** 2020-05-04

**Authors:** Erik D. Fagerholm, W. M. C. Foulkes, Yasir Gallero-Salas, Fritjof Helmchen, Karl J. Friston, Rosalyn J. Moran, Robert Leech

**Affiliations:** 1 Department of Neuroimaging, King’s College London, London, United Kingdom; 2 Department of Physics, Imperial College London, London, United Kingdom; 3 Brain Research Institute, University of Zürich, Zürich, Switzerland; 4 Neuroscience Center Zürich, Zürich, Switzerland; 5 Wellcome Centre for Human Neuroimaging, University College London, London, United Kingdom; Newcastle University, UNITED KINGDOM

## Abstract

In contrast to the symmetries of translation in space, rotation in space, and translation in time, the known laws of physics are not universally invariant under transformation of scale. However, a special case exists in which the action is scale invariant if it satisfies the following two constraints: 1) it must depend upon a scale-free Lagrangian, and 2) the Lagrangian must change under scale in the same way as the inverse time, 1t. Our contribution lies in the derivation of a generalised Lagrangian, in the form of a power series expansion, that satisfies these constraints. This generalised Lagrangian furnishes a normal form for dynamic causal models–state space models based upon differential equations–that can be used to distinguish scale symmetry from scale freeness in empirical data. We establish face validity with an analysis of simulated data, in which we show how scale symmetry can be identified and how the associated conserved quantities can be estimated in neuronal time series.

## Author summary

Considerations of the way in which a dynamical system changes under transformation of scale offer insight into its operational principles. Scale freeness is a paradigm that has been observed in a variety of physical and biological phenomena and describes a situation in which appropriately scaling the space and time coordinates of any evolution of the system yields another possible evolution. In the brain, scale freeness has drawn considerable attention, as it has been associated with optimal information transmission capabilities. Scale symmetry describes a special case of scale freeness, in which a system is *perfectly* unchanged under transformation of scale. Noether’s theorem tells us that in a system that possesses such a symmetry, an associated conservation law must also exist. Here we show that scale symmetry can be identified, and the related conserved quantities measured, in both simulations and real-world data. We achieve this by deriving a generalised equation of motion that leaves the action invariant under spatiotemporal scale transformations and using a modified version of Noether’s theorem to write the associated family of conservation laws. Our contribution allows for the first such statistical characterisation of the quantity that is conserved purely by virtue of scale symmetry.

## Introduction

A symmetry is a transformation to a physical law that leaves its mathematical form invariant [1]. For instance, the known laws of physics are invariant under translation in space, rotation in space, and translation in time. In other words: having taken all influencing factors into account, it is impossible for an external observer to determine whether a dynamical system has been shifted to a new location, rotated by a fixed angle, or whether its onset has been shifted in time.

However, the laws are generally not invariant under transformation of scale. Richard Feynman famously described an intuitive example of why this is the case for a scale transformation within a gravitational field. He asked the audience to consider a thought experiment in which an intricate cathedral made of matchsticks was increased in size to the point where it would instead be made of great logs, thus collapsing under its own weight. The scale dependence of this system is further emphasized by his observation that:

*“…when you’re comparing two things you must change everything that’s in the system. The little cathedral made with matchsticks is attracted to the Earth. So, to make the comparison I should make the big cathedral attracted to an even bigger Earth*. *Too bad–a bigger Earth would attract it even more and the sticks would break even more surely.” [2]*

Scale symmetries are therefore not universally applicable in the same way as translation in space, rotation in space, and translation in time. However, there are known constraints (see Materials and Methods) under which scale symmetries *can* arise in dynamical systems.

In 1918 Noether demonstrated that for every continuous symmetry of the action of a dynamical system there exists a corresponding conservation law [3]. This theorem tells us that it is by virtue of the symmetries of translation in space, rotation in space, and translation in time that the corresponding quantities of linear momentum, angular momentum, and energy are conserved, respectively.

It is the purpose of the present work to devise a method for estimating scale symmetries and their associated conserved quantities in empirical time series.

Materials and Methods are presented in two sections:

In the first section, we introduce the principle of stationary action, the distinction between scale freeness and scale symmetry, and Noether’s Theorem. We then show that an equation of motion leads to a scale invariant action under the constraints that its Lagrangian: 1) is scale-free, and 2) transforms inversely with time under change of scale.

In the second section, the main contribution of this paper is presented via the derivation of a generalised scale-symmetric Lagrangian, in the form of a power series expansion, which can be used to model time series from any scale-free system that follows the principle of stationary action. We then use Noether’s theorem to write the expression for the family of conservation laws associated with this generalised Lagrangian.

Results are presented in two sections:

In the first section, we demonstrate proof of principle by showing that the generalised Lagrangian can be used to distinguish scale symmetry from scale freeness via simulations of a classical particle.

In the second section, using murine calcium imaging and macaque monkey fMRI datasets, we show that neural systems support a neurobiologically-based quantity that is conserved by virtue of scale symmetry.

## Results

See Materials and Methods for all definitions, techniques, and equations.

**Fig 1:**
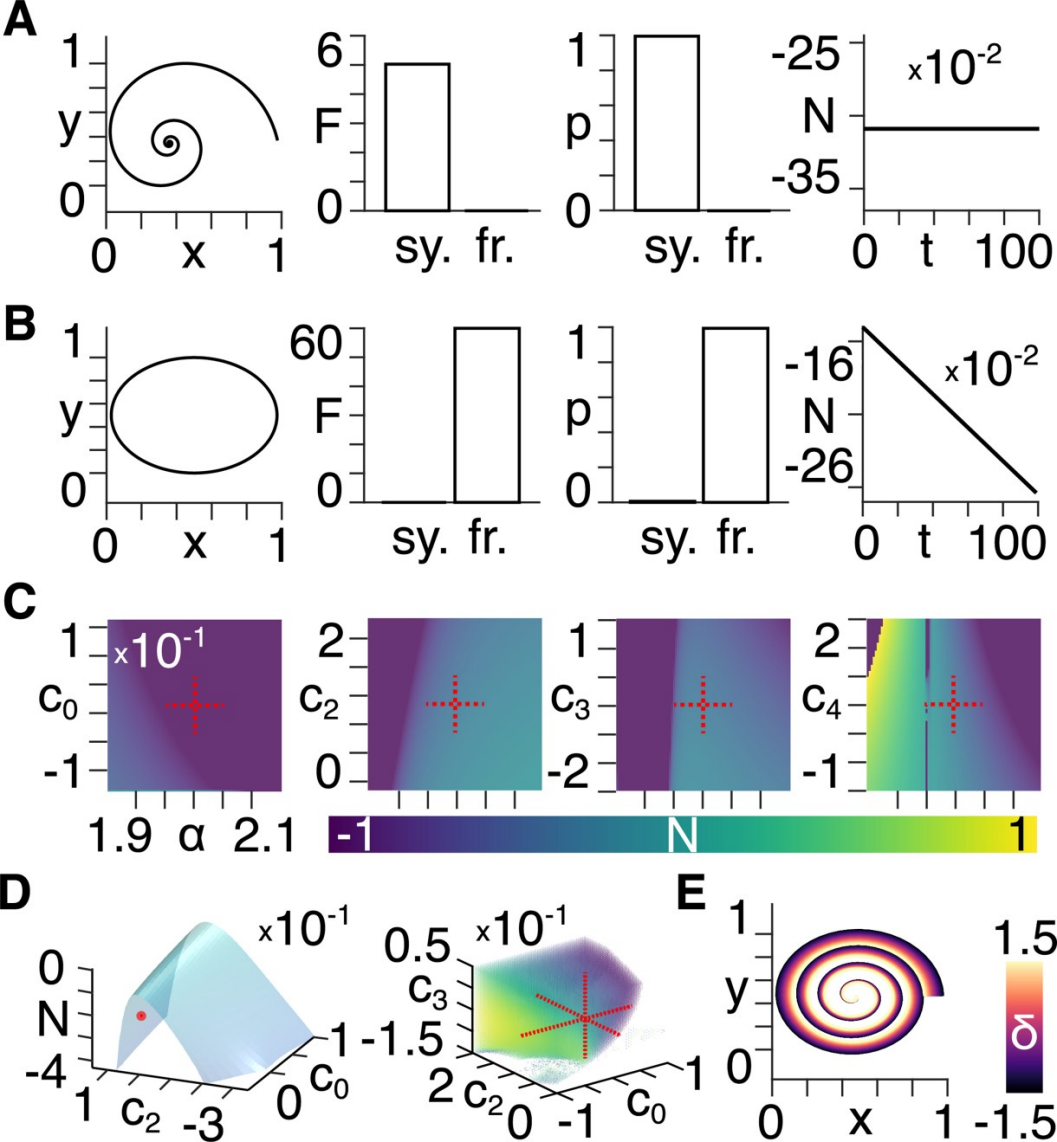
Simulations of a classical particle. **A)** In order from left to right: 1) the trajectory of a particle moving under the influence of a force that varies inversely as the cube of position; 2) Approximate lower bound log model evidence given by the free energy (F) following Bayesian model reduction for scale-symmetric (sy.) and scale-free (fr.) models; 3) Probabilities (p) derived from the log evidence; 4) Noether conserved quantity (N) as a function of time with low noise; **B)** Same layout as A) but for a particle moving under the influence of a force that varies inversely as the square of position; **C)** Noether conserved quantity values between negative and positive unity, as indicated by the colour bar, for the four expansion coefficients (left to right) as a function of α. The centred red cross indicates the posterior densities in A); **D)** Left: Noether conserved quantity as a function of the first two expansion coefficients, with the posterior density values obtained in A) shown by the red dot; Right: Noether conserved quantity as a function of the first three expansion coefficients, with the posterior densities obtained from A) indicated by the centred red cross; **E)** The equation of motion resulting from a forward generative model for different values of δ as indicated by the colour bar.

We use two ground truth datasets in the form of (noiseless) particle trajectories that are known *a priori* to be a) scale-symmetric, and b) scale-free. In Eqs (28) through (30) we show that the scale-symmetric case arises when the particle experiences a force that varies inversely as the cube of its distance from the origin, which in turn is known to result in an logarithmic spiral trajectory [4] (Fig 1A, left). In the scale-free case we use an inverse square force law which is known to result, for instance in planetary orbits, in an elliptical trajectory [5] (Fig 1B, left). Note that we use a version of (34) in which we accommodate both an *x* and *y* coordinate, as shown in the accompanying code, to allow for the particles to trace 2D trajectories (see S1 Text). This means that the conserved quantity in (23) can be related directly to the geometric properties of the logarithmic spiral in the scale-symmetric case (e.g. the polar slope and curvature).

We use Dynamic Expectation Maximisation (DEM) [6] to infer the latent states and estimate the parameters (and hyperparameters; i.e. the precision components of random fluctuations on the states and observation noise). Having applied the optimization to the full model comprising a non-zero *δ* (i.e. scale-free) in Eq (33) we subsequently use Bayesian model reduction [7,8] to estimate the evidence for the reduced model in which *δ* = 0 (i.e. scale-symmetric). We specify the reduced model by setting the prior variance over the *δ* parameter to zero, where *δ* is also given a prior mean of zero.

**Fig 2:**
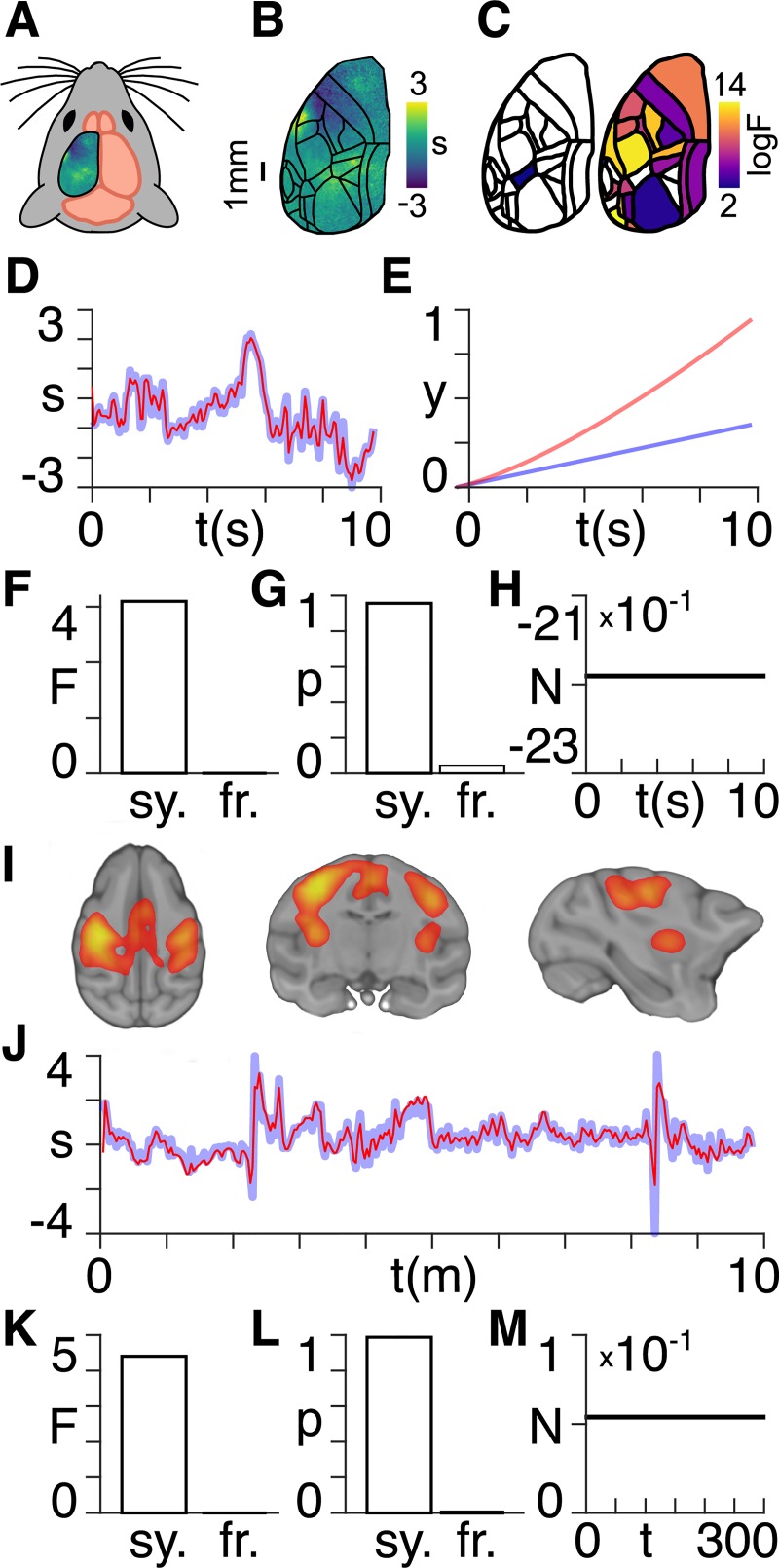
Neuroimaging data. **A)** Wide-field calcium imaging over the left hemisphere of a head-fixed mouse, expressing GCaMP6f in layer 2/3 excitatory neurons; **B)** Example z-scored (DF/F) activity averaged over a 10s trial length, shown as standard deviation (s) of the signal from the mean. Cortical areas are aligned to the Allen Mouse Common Coordinate Framework; **C)** Log variational free energy values corresponding to the colour bar, thresholded at F = 3 for regions found to have higher model evidence for scale symmetry (left) and freeness (right); **D)** z-scored (DF/F) activity shown as standard deviation (s) of the signal from the mean from an example trial in one mouse in the scale-symmetric region (blue), together with the estimated time series following model inversion (red), **E)** Normalized timecourses of observable measurements (y) showing the evolution of the scale-free (blue) and scale-symmetric (red) equations of motion with low noise and without driving inputs; **F)** Approximate lower bound log model evidence given by the free energy (F) following Bayesian model reduction for scale-symmetric (sy.) and scale-free (fr.) models in the calcium imaging data; **G)** Probabilities (p) derived from the log evidence in F); **H)** Noether conserved quantity (N) as a function of time for the calcium imaging data; **I)** The region explaining the highest amount of variance defined via temporal-concatenation probabilistic ICA, thresholded at z>3; **J)** z-scored fMRI activity shown as standard deviation (s) of the signal from the mean from an example scan in the scale-symmetric network (blue), together with the estimated time series following model inversion (red). **K)** Approximate lower bound log model evidence given by the free energy (F) following Bayesian model reduction for scale-symmetric (sy.) and scale-free (fr.) models in the fMRI data; **L)** Probabilities (p) derived from the log evidence in K); **M)** Noether conserved quantity (N) as a function of time for the fMRI data.

Using Bayesian model inversion, followed by model reduction, we show that the correct model is identified (Fig 1A and 1B centre). We subsequently use the posterior expectations of the parameters for the full (scale-free) and reduced (scale-symmetric) models to show that the Noether conserved quantity (or Noether charge) is constant in time for the scale-symmetric (Fig 1A, right), but not for the scale-free case (Fig 1B right). We then explore the way in which the value of the Noether conserved quantity varies within the parameter space close to the posterior densities in terms of 1) *α* vs. each of the expansion coefficients (Fig 1C); 2) the first two expansion coefficients (Fig 1D, left); and 3) the first three expansion coefficients (Fig 1D, right), with a rotating version shown in S1 Movie. Finally, we run the model forward to show the behaviour of the pure equation of motion in the *δ* parameter range −1.5<*δ*<1.5, thus showing the transition from scale freeness with *δ*<0, through scale symmetry (*δ* = 0), and back to scale freeness with *δ*>0 (Fig 1E).

### Neuroimaging data

Here, we analyse murine calcium imaging [9] (rest and task) and macaque monkey fMRI [10] (rest and anaesthetised) datasets, using the same techniques as with the particle simulations described above. The fMRI datasets are taken from the Nathan Kline Institute Macaque Dataset 1, in which twelve fMRI scans (each approximately 10 minutes long) are acquired in a single monkey in an awake state and twelve in an anaesthetized state. The macaque monkey was sedated with dexdomitor (0.02 mg/kg IM), ketamine (8 mg/kg IM), atropine (0.05 mg/kg IM), and maintained at an isoflurane level of 0.75% following intubation. Pre-processing of the murine calcium imaging [11] and macaque monkey fMRI [12] datasets were carried out as described previously. We show all results obtained for the resting states in Fig 2.

The calcium imaging data were collected across an entire hemisphere of mouse cortex (Fig 2A & 2B). We perform Bayesian model averaging across *n* = 3 mice with 10 trials of 10s (200 time points) duration each. We find that there is higher model evidence for scale symmetry, as opposed to scale freeness, in a single region (Fig 2C, left). All other regions show either higher model evidence for scale freeness, or else cannot be statistically classified either as scale-symmetric or scale-free–these regions are not coloured and appear white (Fig 2C, right). No region emerges as scale-symmetric in the task state. We show a sample timecourse from the region classified as scale-symmetric, together with the estimated data following model inversion (Fig 2D). We then run both the full (scale-free) and reduced (scale-symmetric) models forward, with low noise in the absence of external inputs, in order to show the way in which the pure equations of motion evolve in time (Fig 2E). We show the variational free energy (Fig 2F) and associated probability (Fig 2G) of the reduced model for the scale-symmetric region and run the model forward, with parameters furnished by posterior densities from the scale-symmetric model to show that Noether’s conserved quantity N is constant in time (Fig 2H).

In the macaque monkey fMRI data, we observe higher model evidence for scale symmetry, as opposed to scale freeness, in a single cortical network (Fig 2I). We show a sample timecourse, together with the estimated data following model inversion, from this scale-symmetric network (Fig 2J). We also calculate the variational free energy (Fig 2K), associated probabilities (Fig 2L), and Noether conserved quantity (Fig 2M) for this scale-symmetric network. No network emerges as scale-symmetric in the anaesthetised state.

Note that, although we focus on neuroimaging data in this study, these tools can be used to distinguish between scale symmetry and scale freeness in any dynamical system with measurable time series without restriction upon data dimensionality–provided that the system: a) operates with scale-free dynamics and b) follows the principle of stationary action.

## Discussion

In contrast to the symmetries of translation in space, rotation in space, and translation in time, the known laws of physics are not universally invariant under transformation of scale. In fact, as we show in Eqs (28) to (30) (see Materials and Methods), the only way for a classical 1D time-independent Lagrangian to qualify as scale-symmetric is if its potential energy term varies as the inverse square of position. More generally, we show that a scale-free dynamical system that follows the principle of stationary action is only scale-symmetric in the special case that its Lagrangian scales inversely with time (see Eq (12) in Materials and Methods).

This restrictive condition may explain why symmetries under change of scale are not usually discussed in the context of dynamical systems. Another reason could be that a symmetry is often defined as being contingent on a Lagrangian remaining invariant, which would only be possible in a scale transformation if the rescaling factors preceding the spatial and temporal variables cancelled each other in every term. However, such a definition of scale symmetry would only be compatible with Noether’s theorem if the Jacobian associated with the rescaling of the temporal variable were equal to unity. In the case of a non-unity Jacobian, quantities conserved by virtue of scale symmetry only exist if one redefines what is meant by scale symmetry to include a factor that cancels the Jacobian. No other definition leads to a conservation law. In other words, instead of satisfying the sufficient but not necessary condition of an invariant Lagrangian, we allow for the existence of scale symmetry via the sufficient *and* necessary condition of an invariant action.

To demonstrate the practical applicability of the theoretical results, we derive an expression for a generalised scale-symmetric Lagrangian in the form of a power series expansion (see Eq (16) in Materials and Methods) and show that this can be used to distinguish scale symmetry from scale freeness in ground truth models of classical particle trajectories. We then use Noether’s theorem to write the family of conservation laws that arise under change of scale for this generalised scale-symmetric Lagrangian. Assuming that neural systems operate with scale-free dynamics [13–15] and evolve via a stationary action principle [16–18], we therefore establish a link between scaling properties and conservative aspects of neuronal message passing (e.g. excitation/inhibition balance [19,20])–two fields that have thus far largely been studied in isolation in neuroscience. When describing angular momentum one can turn to familiar real-world examples involving e.g. an ice skater spinning faster upon retracting her arms. Yet, if asked to provide a similarly intuitive understanding of the quantity conserved by virtue of scale symmetry, we would be hard-pressed. We can, however, attempt to better understand this quantity by mapping the way in which it varies with respect to different parameters (see Fig 1C–1E & S1 Movie).

In summary, our demonstration makes use of a generative model, allowing for the assessment of scale freeness/symmetry directly from neuroimaging data collected at a single spatial and temporal scale. We build an inference tool that allows for nonlinear effects with complex noise to be identified in the context of a hypothesised scale free dynamical systems architecture. Crucially, this solution to the inverse problem (from data to mechanism) enables us to test for alternate scaling principles. We hope that our theoretical framework, as well as the data and code we have made publicly available, will allow researchers to apply this methodology across a broader range of datasets, in order to reveal clues as to the biological underpinning of conservation laws arising by virtue of scale symmetries in neural systems

## Materials and methods

### The principle of stationary action

In the Lagrangian formulation of classical mechanics, the evolution of a dynamical system along a trajectory from an initial time *t*_*i*_ to a final time *t*_*f*_ is associated with a number, known as the action, which is calculated by integrating the Lagrangian function of the system’s position and velocity along the trajectory. The action can be evaluated for any trajectory, but trajectories that satisfy the equation of motion and thus might be followed in reality are distinguished because they render the action stationary. That is, a small variation of any trajectory that satisfies the equation of motion leaves the value of the action unchanged to first order [21]. This principle of stationary action, also known as Hamilton’s principle, is a powerful mathematical tool for investigating dynamical systems and has found ubiquitous use in the physical sciences. Almost all of modern physics, including field-theoretic descriptions of electromagnetism, gravity and quantum theory, can be re-cast in terms of the principle of stationary action.

In this work, we consider a Lagrangian with explicit time-dependence $L(q,\dot{q},t)$ to facilitate the analysis of driven systems. The principle of stationary action tells us that the trajectory *q*(*t*) followed by the system from any chosen initial point *q*_*i*_ at time *t*_*i*_ to any chosen final point *q*_*f*_ at time *t*_*f*_ renders the action, given by:

$$S[q(t)]=\int_{t_{i}}^{t_{f}}L(q(t),\frac{dq(t)}{dt},t)dt,$$ (1)

stationary.

In other words, for any infinitesimal path variation *δq*(*t*) satisfying *δq*(*t*_*i*_) = *δq*(*t*_*f*_) = 0, we must have:

$$S[q(t)+\delta q(t)]=S[q(t)]+O[{\left(\delta q\right)}^{2}].$$ (2)

One can then use standard arguments [22] to show that any trajectory *q*(*t*) for which the action is stationary is a solution of the Euler-Lagrange equation:

$$\frac{\partial L}{\partial q}-\frac{d}{dt}\left(\frac{\partial L}{\partial \dot{q}}\right)=0.$$ (3)

### Scale freeness

Scale freeness describes a situation in which different levels of magnification of a dynamical system are indistinguishable to within a multiplicative constant [23]. Given the set of points (*t*,*q*) lying on some chosen trajectory *q*(*t*), we define the corresponding scaled trajectory as the set of points (*t*_*s*_,*q*_*s*_) = (*λ*^*α*^*t*,*λq*), where *λ* (>0) is a spatial scale factor and the time coordinate has been rescaled by *λ*^*α*^, where *α* is a system-dependent constant.

The scaled trajectory passes through the point:

$$\begin{array}{c} q_{s}=\lambda q,\\ t_{s}=\lambda^{\alpha }t, \end{array}$$ (4)

implying that *q*_*s*_(*λ*^*α*^*t*) = *λq*(*t*), or, equivalently that:

$$q_{s}(t_{s})=\lambda q(\lambda^{-\alpha }t_{s}),$$ (5)

from which it follows that:

$$\frac{dq_{s}(t_{s})}{dt_{s}}=\lambda^{1-\alpha }\dot{q}(\lambda^{-\alpha }t_{s}).$$ (6)

We refer to the system’s dynamics as being scale-free if, for any path *q*(*t*):

$$S[q(t)]=\kappa \;S[q_{s}(t_{s})],$$ (7)

where *κ* is a constant that may depend on the scale factor *λ* but is independent of path.

More explicitly, using (1) and (7), we see that the system is scale-free if:

$$\int_{t_{i}}^{t_{f}}L(q(t),\frac{dq(t)}{dt},t)dt=\kappa \int_{\lambda^{\alpha }t_{i}}^{\lambda^{\alpha }t_{f}}L(q_{s}(t_{s}),\frac{dq_{s}(t_{s})}{dt_{s}},t_{s})dt_{s}.$$ (8)

Assuming that *q*(*t*) is a physical trajectory derived by applying the principle of stationary action to a scale-free action, it follows from (2) and (7) that:

$$S[q_{s}(t_{s})+\delta q_{s}(t_{s})]=S[q_{s}(t_{s})]+O[{\left(\delta q_{s}\right)}^{2}],$$ (9)

for all infinitesimal path variations *δq*_*s*_(*t*_*s*_).

This shows that the scaled path described by (5) also renders the action stationary, i.e. if *q*(*t*) is a possible physical trajectory then the same can be said for the scaled trajectory *q*_*s*_(*t*_*s*_) = *λq*(*λ*^−*α*^*t*_*s*_).

Scale freeness has been observed in a variety of physical and biological settings [24]. These include neural systems across different species [25,26], in which evidence for scale freeness is identified by signatures of critical neuronal dynamics [27,28], and is considered to offer functional [29], developmental [30], as well as evolutionary [31,32] advantages. However, some studies recognize the lack of sufficient orders of magnitude in spatial and temporal scale within such studies in neuroscience [33]. Furthermore, there are known limitations inherent in indirectly inferring scale freeness on the basis of proximity to power law behavior in neural cascading events [34] or in power frequency plots [35].

### Scale symmetry

We say that a system is scale-symmetric if it is impossible to determine the magnification at which its evolution is observed. In other words, scale symmetry means that a system is *perfectly* unchanged under transformation of scale, i.e. by setting *κ* = 1 in Eq (8), such that:

$$\int_{t_{i}}^{t_{f}}L(q(t),\frac{dq(t)}{dt},t)dt=\int_{\lambda^{\alpha }t_{i}}^{\lambda^{\alpha }t_{f}}L(q_{s}(t_{s}),\frac{dq_{s}(t_{s})}{dt_{s}},t_{s})dt_{s}.$$ (10)

### The condition for scale symmetry

We see via (5), (6) and (10) that:

$$\begin{array}{l} \int_{t_{i}}^{t_{f}}L(q(t),\dot{q}(t),t)dt=\int_{\lambda^{\alpha }t_{i}}^{\lambda^{\alpha }t_{f}}L(\lambda q({\lambda^{-\alpha }t}_{s}),\lambda^{1-\alpha }\dot{q}(\lambda^{-\alpha }t_{s}),t_{s})dt_{s}\\ \;=\lambda^{\alpha }\int_{t_{i}}^{t_{f}}L(\lambda q(t),\lambda^{1-\alpha }\dot{q}(t),\lambda^{\alpha }t)dt, \end{array}$$ (11)

where, using (4), the integration variable on the right-hand side was changed from *t*_*s*_ to *t* = *λ*^−*α*^*t*_*s*_.

Since the path of integration is arbitrary, it follows that the action is scale-symmetric if and only if the Lagrangian satisfies:

$$L_{s}(q,\dot{q},t)\equiv L(\lambda q,\lambda^{1-\alpha }\dot{q},\lambda^{\alpha }t)=\lambda^{-\alpha }L(q,\dot{q},t),$$ (12)

where the identity defines the scaled Lagrangian $L_{s}$ and the equality describes the condition for scale symmetry.

We therefore see that scale symmetry can exist in scale-free systems if these can be described by a Lagrangian that scales inversely with time. In other words, given a spatiotemporal transformation in which *q*→*λq* and *t*→*λ*^*α*^*t*, the system is scale-symmetric if the Lagrangian transforms as $L\to \lambda^{-\alpha }L$, implying that L scales in the same way as 1t.

### A family of scale-symmetric Lagrangians

Here, we present the main contribution of this paper via the derivation of a generalised scale-symmetric Lagrangian that can be used to identify scale symmetry in time series from any scale-free dynamical system that follows the principle of stationary action.

We can write an expression for a Lagrangian $L(q,\dot{q},t)$ as a sum over power terms:

$$L(q,\dot{q},t)=\sum_{x,y,z}C_{xyz}q^{x}{\dot{q}}^{y}t^{z},$$ (13)

where *x*, *y* and *z* are constants and *C*_*xyz*_ is an arbitrary expansion coefficient.

Using (12) we see that (13) is scale-symmetric if:

$$L_{s}(q,\dot{q},t)=L(\lambda q,\lambda^{1-\alpha }\dot{q},\lambda^{\alpha }t)=\sum_{x,y,z}\lambda^{x+(1-\alpha )y+\alpha z}C_{xyz}q^{x}{\dot{q}}^{y}t^{z}=\lambda^{-\alpha }L(q,\dot{q},t),$$ (14)

and since *λ* is arbitrary, this implies that:

x+(1−α)y+αz=−α, (15)

∀ *x*,*y*,*z*: *C*_*xyz*_≠0.

We can use (15) to uniquely determine the value of *x* given *α* and the knowledge that a non-zero term with specific values of *y* and *z* exists. This in turn means that we can replace the triple summation in (13) with a double summation:

$$L(q,\dot{q},t)=q^{-\alpha }\sum_{y,z}C_{yz}q^{y(\alpha -1)-z\alpha }{\dot{q}}^{y}t^{z},$$ (16)

which describes a family of scale-symmetric Lagrangians.

We arrive in (16) at a general Lagrangian that satisfies the condition for scale symmetry for a system with (*z*≠0) or without (*z* = 0) external driving inputs. This means that one can use an expansion of this expression (to the desired number of terms) as a forward generative model. This model is capable of creating data and also of applying an inverse procedure (fitting) to recover key model parameters from arbitrary time series. This can be done for any dynamical system (i.e., not necessarily neural systems), in order to determine the extent to which they can be approximated by a scale-symmetric function. Note that one can in principle restrict the allowed values of the exponents in (16) to be natural numbers in order to obtain an analytic function. However, for the purpose of the work presented here we do not place such a restriction, in order to allow for greater flexibility in subsequent time series analyses.

### Noether’s theorem and scale symmetry

Beginning from the statement of scale symmetry (11), we set *λ* = 1+*ϵ*, where *ϵ* is an arbitrarily small constant. This allows for any scale transformation to be constructed by sequentially applying such infinitesimal transformations.

Working to first order in *ϵ* we can write (11) as follows:

$$\int_{t_{i}}^{t_{f}}L(q,\dot{q},t)dt=\int_{t_{i}}^{t_{f}}L((1+\epsilon )q,(1+(1-\alpha )\epsilon )\dot{q},(1+\alpha \epsilon )t)(1+\alpha \epsilon )dt.$$ (17)

Expanding the right-hand side and cancelling the *ϵ*-independent terms we see that:

$$\epsilon \int_{t_{i}}^{t_{f}}\{q\frac{\partial L}{\partial q}+(1-\alpha )\dot{q}\frac{\partial L}{\partial \dot{q}}+\alpha t\frac{\partial L}{\partial t}+\alpha L\}dt=0,$$ (18)

and since $\frac{dL}{dt}=\frac{\partial L}{\partial t}+\frac{\partial L}{\partial q}\dot{q}+\frac{\partial L}{\partial \dot{q}}\ddot{q}$, this is equivalent to:

$$\epsilon \int_{t_{i}}^{t_{f}}\{q\frac{\partial L}{\partial q}+(1-\alpha )\dot{q}\frac{\partial L}{\partial \dot{q}}+\alpha L+\alpha t(\frac{dL}{dt}-\frac{\partial L}{\partial q}\dot{q}-\frac{\partial L}{\partial \dot{q}}\ddot{q})\}dt=0.$$ (19)

If we now stipulate that *q*(*t*) is a physical path of the system, we can use the Euler-Lagrange Eq (3) to eliminate $\frac{\partial L}{\partial q}$ from (19) in order to obtain:

$$\epsilon \int_{t_{i}}^{t_{f}}\{(q-\alpha t\dot{q})\frac{d}{dt}(\frac{\partial L}{\partial \dot{q}})+(1-\alpha )\dot{q}\frac{\partial L}{\partial \dot{q}}+\alpha L+\alpha t\frac{dL}{dt}-\alpha t\ddot{q}\frac{\partial L}{\partial \dot{q}}\}dt=0,$$ (20)

which can be rewritten as:

$$\epsilon \int_{t_{i}}^{t_{f}}\frac{d}{dt}\left\{(q-\alpha t\dot{q})\frac{\partial L}{\partial \dot{q}}+\alpha tL\right\}dt=0,$$ (21)

from which we see that the quantity:

$$N=(q-\alpha t\dot{q})\frac{\partial L}{\partial \dot{q}}+\alpha tL=\frac{\partial L}{\partial \dot{q}}q-H\alpha t,$$ (22)

must have the same value at the (arbitrary) initial and final times *t*_*i*_ and *t*_*f*_, where the total energy, or Hamiltonian, $H=\dot{q}\frac{\partial L}{\partial \dot{q}}-L$.

We therefore arrive at a special case of Noether’s theorem in (22) applicable to scale-symmetric systems.

### Conservation laws associated with a family of scale-symmetric Lagrangians

**Fig 3:**
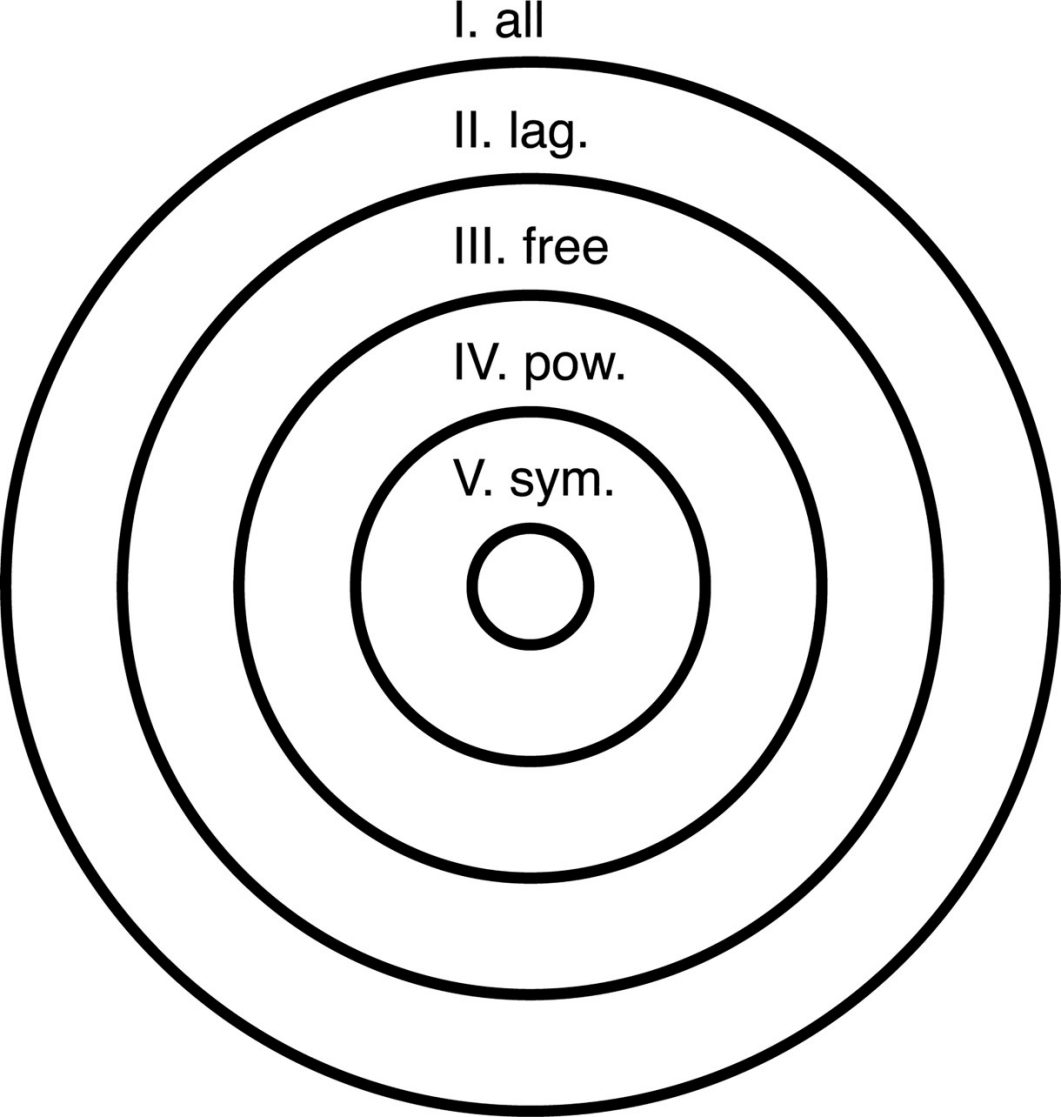
Scale-symmetric functions. In order of decreasing size, the areas of the circles represent the space of I. All possible functions; II. Functions that can be cast within a Lagrangian framework; III. Scale-free Lagrangians, IV. Scale-free Lagrangians that can be expressed as a power series; and V. Scale-symmetric power series Lagrangians. It is for this smallest subset of functions for which the quantities in (23) are conserved by virtue of scale symmetry.

Using (22) we can now write an expression for the conserved quantities associated with the family of scale-symmetric Lagrangians in (16):

$$N=\alpha q^{-\alpha }t\sum (1-y)C_{yz}q^{y(\alpha -1)-z\alpha }{\dot{q}}^{y}t^{z}+q^{1-\alpha }{\dot{q}}^{-1}\sum yC_{yz}q^{y(\alpha -1)-z\alpha }{\dot{q}}^{y}t^{z}.$$ (23)

which arise within a specific subset of functions (Fig 3):

We therefore arrive in (23) at an expression for all possible conserved quantities that arise in a dynamical system that possesses a symmetry under transformation of scale, i.e. one that can be modelled by (16).

### Free classical particle

Here we analyse what is perhaps the simplest example of a dynamical system, in the form of a classical particle moving in the absence of applied forces. The motion of this free particle is described by the following Lagrangian:

$$L=\frac{1}{2}m{\dot{q}}^{2},$$ (24)

which, using (6), transforms under scale as follows:

$$L\to L_{s}=\lambda^{2(1-\alpha )}\frac{1}{2}m{\dot{q}}^{2}.$$ (25)

This satisfies the condition for scale symmetry in (12) when:

2(1−α)=−α⟹α=2, (26)

which, together with (22) and (24), shows us that the corresponding conserved quantity is given by:

$$N=\left(\frac{1}{2}m{\dot{q}}^{2}-m{\dot{q}}^{2}\right)2t+mq\dot{q}=m\dot{q}(q-\dot{q}t).$$ (27)

Since, for a free particle, $\dot{q}$ is constant and $q=q_{i}+\dot{q}(t-t_{i})$, we see that *N* is indeed conserved along the trajectory.

### Classical particle in a potential

We now consider the effect of adding a potential energy term to (24), such that the Lagrangian is given by:

$$L=\frac{1}{2}m{\dot{q}}^{2}+kq^{p},$$ (28)

where *k* and *p* are constants.

Using (4) and (6) we see that (28) transforms under scale as:

$$L\to L_{s}=\lambda^{2(1-\alpha )}\frac{1}{2}m{\dot{q}}^{2}+\lambda^{p}kq^{p}.$$ (29)

From (12) we know that a scale symmetry exists if and only if $L_{s}=\lambda^{-\alpha }L$, implying that 2(1−*α*) = *p* = −*α* and hence that *α* = 2 and *p* = −2.

To be scale-symmetric, the potential must therefore be an inverse square and the Lagrangian in (28) must take the following form:

$$L=\frac{1}{2}m{\dot{q}}^{2}+kq^{-2}.$$ (30)

In other words, in order for a particle described by (28) to be scale-symmetric, it must be acted upon by a force that varies inversely as the cube of its distance from the origin–a special case that has been analysed previously [36,37]. We include a 2D generalization of Eqs (28) to (30) in S1 Text, as is used for the particle simulations in Fig 1.

Using (22) and (30) we see that:

$$N=mq\dot{q}-(m{\dot{q}}^{2}-2kq^{-2})t,$$ (31)

which can be simplified by noting from (30) that the system’s total energy, or Hamiltonian, is given by $H=\frac{1}{2}m{\dot{q}}^{2}-kq^{-2}$, which means that (31) can be re-written as:

$$N=mq\dot{q}-2Ht.$$ (32)

One can then use Newton’s second law: $m\ddot{q}=-2k/q^{3}$, together with (32), to verify that $\frac{dN}{dt}=0$. We therefore arrive at expression in (32) for the quantity that is conserved by virtue of scale symmetry for a classical particle moving under the influence of an inverse-cube force law.

### Classical particle simulations

Here we use Bayesian model inversion, followed by model reduction, to demonstrate face validity by using Dynamic Causal Modelling (DCM) [38] to distinguish between datasets that are known to be a) scale-symmetric; and b) scale-free but not scale-symmetric—henceforth referred to simply as scale-free.

We expand (16) to fifth order for a system with a time-independent Lagrangian and multiply the resultant expression by *q*^*δ*^, where *δ* is a constant, such that:

$$L=q^{-\alpha +\delta }\sum_{y=0}^{4}C_{y}q^{y(\alpha -1)}{\dot{q}}^{y},$$ (33)

thus allowing us to use *δ* a measure of deviation from scale symmetry, i.e. we can use (33) to describe the two cases in which the system is a) scale-symmetric when *δ* = 0; and b) scale-free when *δ*≠0. Note that we have now re-defined *α* as the exponent required for the Lagrangian to be scale-symmetric.

We then use the Euler-Lagrange Eq (3) to recover the equation of motion associated with (33), to which we apply noise terms describing random, non-Markovian fluctuations [39] within the Statistical Parametric Mapping (SPM) software. This means that we use (33) as a state space model of observable measurements *y* by equipping the associated equations of motion with random fluctuations *ω*_*f*_ and mapping the (latent) states to observable quantities with additive observation noise *ω*_*g*_:

$$\begin{array}{l} x=\dot{q}+\omega_{f}^{\left(x\right)}\\ \dot{x}=\frac{q^{-1}\sum_{y=0}^{4}C_{y}(1-y)((\alpha -1)y+\delta -\alpha )q^{(\alpha -1)y}{\dot{q}}^{y}}{\sum_{y=2}^{4}y(y-1)C_{y}q^{(\alpha -1)y}{\dot{q}}^{y-2}}+\omega_{f}^{\left(\dot{x}\right)}\\ y=q+\omega_{g.} \end{array}$$ (34)

This furnishes a dynamic causal model in the form of a stochastic differential equation (where the random fluctuations are assumed to be small). This is the form of the equation of motion used for all analyses presented in this paper. Crucially, the parameters *θ*_*f*_ = (*α*,*δ*,*C*_0_,*C*_2_,*C*_3_,*C*_4_) of this model can now be recovered from observations under the prior assumptions that the underlying dynamics take the form in (34). The equation of motion in (34) can be regarded as a normal form for scale-free systems that becomes scale-symmetric when *δ* = 0. This distinction affords the opportunity to assess the evidence for scale symmetry by comparing models both with and without tight shrinkage priors on *δ*, i.e. determining if *δ* is non-zero.

### Parameter estimation and model comparison

Model inversion is applied to the simulated and empirical datasets in order to estimate the model parameters. We use a variational Bayesian inversion scheme which comprises a gradient ascent on the (negative) variational free energy *F*≈*ln*(*p*(*D*|*m*)), where *D* are the data, and *m* is the model.

In variational schemes, posterior densities over parameters *θ*_*f*_ = (*α*,*δ*,*C*_0_,*C*_2_,*C*_3_,*C*_4_) and hyperparameters $h=\omega_{f}^{\left(x\right)},\omega_{f}^{\left(\dot{x}\right)},\omega_{g}$ in Eq (34) are obtained via an optimisation algorithm. Specifically, we use Dynamic Expectation Maximisation (MATLAB code spm_DEM.m from https://www.fil.ion.ucl.ac.uk/spm/), which uses a mean field partition to obtain gradients for three distinct sets of latents–namely the parameters *θ*_*f*_, hyperparameters *h* and the states $x,\dot{x},y$. This provides a probabilistic interpretation of the deterministic dynamics encoded in Eq (34). Priors on model parameters *θ*_*f*_ and hyperparameters *h* are set to 0 and 1/64, respectively. The objective function *F* comprises a sum of log-likelihoods and Kullback-Leibler divergences describing accuracy and model complexity, respectively. Therefore, every iteration of the DEM algorithm should improve the fit, while retaining the most parsimonious set of parameters.

Finally, for model comparison we use a Bayesian model reduction approach by comparing the evidence for models with and without constraints on *δ*, where the constraints imply scale-symmetric (with *δ* = 0) or scale-free (with *δ*≠0) dynamics. We compare these two models using the log Bayes Factor *F*(*δ* = 0)−*F*(*δ*≠0), which returns the relative evidence for scale symmetry over scale freeness. We then calculate the associated probabilities by normalizing *F*, such that $p(\delta =0)=\frac{F(\delta =0)}{F(\delta =0)+F(\delta \neq 0)}$.

## Ethics statement

All animal experiments were carried out according to the guidelines of the Veterinary Office of Switzerland following approval by the Cantonal Veterinary Office in Zürich.

## Supporting information

S1 Text Derivation of a scale symmetric Lagrangian in 2D for a particle moving in a potential. (DOCX)

S1 Movie Variation of the Noether conserved quantity (indicated by the colour bar) within the parameter space close to the posterior densities, in terms of the first three expansion coefficients (*c*_0_, *c*_2_, *c*_3_) in the generalised equation of motion. (MOV)
