# Supplementary material for: Conservation laws by virtue of scale symmetries in neural systems
Source: PLoS Comput Biol. 2020 May 4;16(5):e1007865. doi: 10.1371/journal.pcbi.1007865 (PMC7224579; doi:10.1371/journal.pcbi.1007865)
Supplement: S1 Text — (DOCX) [file pcbi.1007865.s001.docx]

**S1 Text**

Re-writing equation (28) in two dimensions we obtain:

|  | $\mathcal{L=}\frac{1}{2}m\left( \dot{x}^{2}+\dot{y}^{2} \right)+k\left( x^{2}+y^{2} \right)^{\frac{p}{2}},$ | (s1) |
| --- | --- | --- |

where $k$ and $p$ are constants.

Using (4) and (6) we see that (s1) transforms under scale as:

|  | $\mathcal{L \to}\mathcal{L}_{s}=\lambda^{2\left( 1-\alpha\right)}\frac{1}{2}m\left( \dot{x}^{2}+\dot{y}^{2} \right)+\lambda^{p}k\left( x^{2}+y^{2} \right)^{\frac{p}{2}}.$ | (s2) |
| --- | --- | --- |

From (12) we know that a scale symmetry exists if and only if $\mathcal{L}_{s}=\lambda^{-\alpha}\mathcal{L}$, implying that $2\left( 1-\alpha\right)=p=-\alpha$ and hence that $\alpha=2$ and $p=-2$. To be scale-symmetric, the potential must therefore be an inverse square and the Lagrangian in (s2) must take the form:

|  | $\mathcal{L=}\frac{1}{2}m\left( \dot{x}^{2}+\dot{y}^{2} \right)+k\left( x^{2}+y^{2} \right)^{-1}.$ | (s3) |
| --- | --- | --- |
